# RNA-Seq-based analysis of changes in *Borrelia burgdorferi* gene expression linked to pathogenicity

**DOI:** 10.1186/s13071-014-0623-2

**Published:** 2015-03-13

**Authors:** Qiong Wu, Guiquan Guan, Zhijie Liu, Youquan Li, Jianxun Luo, Hong Yin

**Affiliations:** State Key Laboratory of Veterinary Etiological Biology, Key Laboratory of Veterinary Parasitology of Gansu Province, Key Laboratory of Grazing Animal Diseases MOA, Lanzhou Veterinary Research Institute, Chinese Academy of Agricultural Science, Lanzhou, 730046 China

**Keywords:** Lyme disease, Gene expression, High-throughput sequencing, Transcriptional profile

## Abstract

**Background:**

Lyme disease is a global public health problem caused by the spirochaete *Borrelia burgdorferi*. Our previous studies found differences in disease severity between *B. burgdorferi* B31- and *B. garinii* SZ-infected mice. We hypothesized that genes that are differentially expressed between *Borrelia* isolates encode bacterial factors that contribute to disease diversity.

**Methods:**

The present study used high-throughput sequencing technology to characterize and compare the transcriptional profiles of *B. burgdorferi* B31 and *B. garinii* SZ cultured *in vitro*. Real-time quantitative RT-PCR was used to validate selected data from RNA-seq experiments.

**Results:**

A total of 731 genes were differentially expressed between *B. burgdorferi* B31 and *B. garinii* SZ isolates, including those encoding lipoproteins and purine transport proteins. The fold difference in expression for *B. garinii* SZ versus *B. burgdorferi* B31 ranged from 22.07 to 1.01. Expression of the *OspA*, *OspB* and *DbpB* genes were significantly lower in *B. garinii* SZ compared to *B. burgdorferi* B31.

**Conclusions:**

The results support the hypothesis that global changes in gene expression underlie differences in *Borrelia* pathogenicity. The findings also provide an empirical basis for studying the mechanism of action of specific genes as well as their potential usefulness for the diagnosis and management of Lyme disease.

**Electronic supplementary material:**

The online version of this article (doi:10.1186/s13071-014-0623-2) contains supplementary material, which is available to authorized users.

## Background

*Borrelia burgdorferi* is the causative agent of Lyme disease, the most prevalent tick-borne zoonosis and an important emerging infectious disease in Europe, North America, and Far Eastern countries [1]. The *Borrelia burgdorferi* complex consists of 18 proposed and confirmed genospecies [2]. The obligate parasites are transmitted by ticks of *Ixodes* spp., with disease symptoms and severity varying among *B. burgdorferi* genospecies. *B. garinii* is primarily associated with neuroborreliosis [3], *B. afzelii* with crodermatitis chronic athrophicans [4], and *B. burgdorferi* s.s is the major cause of Lyme arthritis [5].

Despite its small genome, the spirochaetes possess complex cellular machinery for regulating gene and protein expression. *B. burgdorferi* expresses specific subsets of genes throughout its life cycle, both in the arthropod vector and vertebrate host [6,7]. In one study of bacterial protein expression in infected mouse tissues, VlsE, OspC, and decorin-binding protein (Dbp)A were expressed at high levels in joints and dermal tissues, while OspC and DbpA were also detected in the heart [8], demonstrating tissue-specific protein expression. A comparative analysis of protein expression profiles of three strains of *B. burgdorferi* (B31, ND40, and JD-1) demonstrated large differences in the percentage of peptide coverage of proteins [9]. The application of genome, transcriptome, interactome, and immunoproteome analyses can reveal complexities of bacterial physiology and pathogenesis; in addition, the development of massively parallel cDNA sequencing (RNA-seq) techniques is enabling more comprehensive and accurate assessments of eukaryote [10] and prokaryote [11] transcriptomes.

Our previous studies found differences in disease severity between *B. burgdorferi* B31- and *B. garinii* SZ-infected mice, particularly affecting the brain, heart, liver, and spleen tissues [12]. Differential gene expression facilitates spirochaetal survival and promotes disease pathogenesis. In the present study, RNA-seq was employed to compare the transcriptome profiles of *B. burgdorferi* B31 and *B. garinii* SZ isolates during *in vitro* culture. The differences in gene expression profiles between the two species of spirochetes provide insights into disease-specific mechanisms.

## Methods

### Bacterial strains

*B. burgdorferi* B31 and *B. garinii* SZ were used in this study. B31 was purchased from the American Type Culture Collection (Manassas, VA, USA) and had undergone five *in vitro* passages. *B. garinii* SZ was isolated from *Dermacentor* ticks collected in Shangzhi county of Heilongjiang province in China [13]. The strains were cultured in BSK-H medium in a 33°C incubator and observed under a dark-field microscope every other day. Cells were harvested by centrifugation at a speed of 5,000 × *g* during logarithmic phase and washed twice with phosphate buffered saline (PBS).

### RNA isolation

RNA was extracted using TRIzol reagent (Invitrogen). RNA concentration and quality were assessed using a NanoDrop ND-1000 spectrophotometer (Thermo Scientific, Wilmington, DE, USA) and Agilent 2100 Bioanalyzer (Agilent Technologies, Santa Clara, CA, USA). RNA (10 μg) was pooled from three individual cells of each strain and used to construct two cDNA libraries following the mRNA sequencing sample preparation guide (Illumina, San Diego, CA, USA). Paired-end DNA sequencing was carried out in two lanes (one per library) on an Illumina HiSeq 2000 following the manufacturer’s protocol. The 16S and 23S rRNA was removed from total RNA using the MICROBExpresst Bacterial mRNA Purification Kit (Ambion, Foster City, CA, USA) according to the manufacturer’s protocol.

### Sequence assembly and annotation

The 100-bp paired-end Illumina reads from the *B. burgdorferi* B31 (82,056,756 reads) and *B. garinii* SZ (145,680,918 reads) libraries were combined for *de novo* assembly. Reads that were of low quality (≥80% with Phred score < 20) or complexity (>80% with single, di-, or trinucleotide repeats) or were < 20 bp were removed. The processed reads were then assembled using the CLC Genomics Workbench v.5.5 [14,15] with wordsize = 45 and minimum contig length ≥ 200. The resulting assembled sequences and singletons were combined and processed to remove duplicates using a custom Perl Script; contigs were then assembled using CAP3 EST to obtain the final unigenes.

Functional annotation of unigenes was achieved by searching for analogous sequences in EMBL and Swiss-Prot databases using an E-value ≤ 1e − 5. Hierarchical functional categorization for gene ontology (GO) terms was accomplished using BLAST2GO, which was also used to identify genes represented among the Kyoto Encyclopedia of Genes and Genomes (KEGG) pathways.

### Real-time quantitative reverse transcription (qRT)-PCR

Real-time qRT-PCR was used to validate data from RNA-seq experiments. Gene-specific primers (Additional file 1: Table S7) were designed using Primer Express software (Applied Biosystems, Carlsbad, CA, USA). The relative quantitation (ΔΔCt) method was used to evaluate differences between the two genospecies for each gene examined. The *fla*B amplicon was used as an internal control to normalize all data. Removal of genomic DNA and reverse transcription (Takara Bio Inc., Otsu, Japan) were performed for each sample and standard without reverse transcriptase to confirm the absence of genomic DNA.

## Results and discussion

Whole-transcriptome profiling of bacteria has been widely used to evaluate global changes in gene expression [16]. RNA-seq-based transcriptome analyses of pathogens during infection yields a robust, sensitive, and accessible dataset that enables the assessment of the regulatory interactions driving pathogenesis [11]. Our previous studies revealed differences in disease severity between *B. burgdorferi* B31- and *B. garinii* SZ-infected mice [12]; the present study used RNA-seq to determine the transcriptional profiles of *B. burgdorferi* B31 and *B. garinii* SZ isolates during *in vitro* infection. This is the first comprehensive analysis of gene expression in this organism; the findings are discussed in the context of pathogenesis, diagnosis, and management of Lyme disease.

### Sequence assembly and annotation

**Figure 1:**
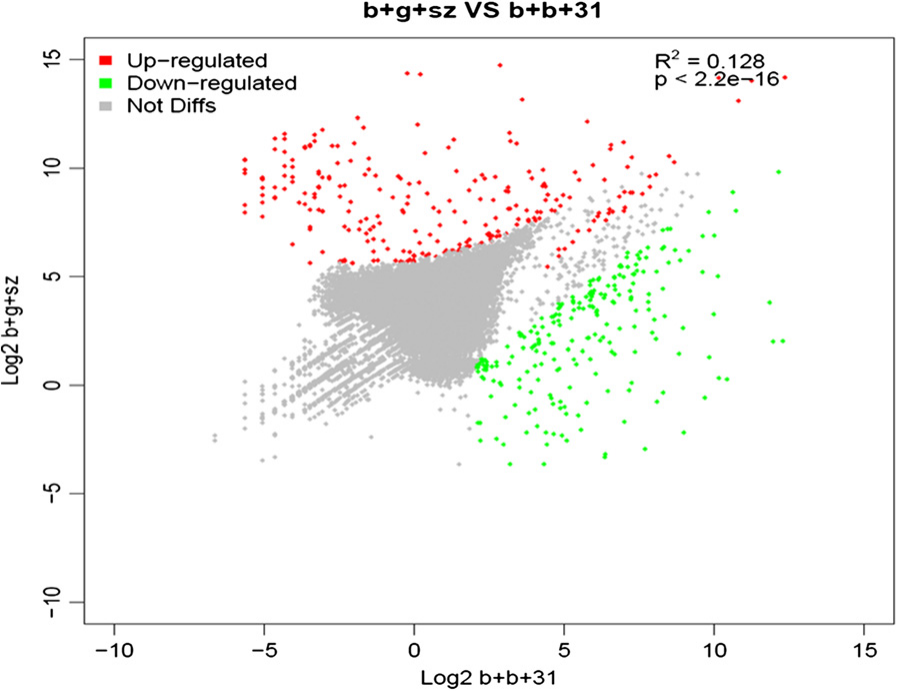
**Average log**_**2**_ **-transformed reads per kilobase per million of genes differentially expressed by** ***B. garinii*** **SZ (y-axis) and** ***B. burgdorferi*** **B31 (x-axis).** Red and green dots represent genes that are significantly up- and downregulated, respectively, in *B. garinii* SZ; gray dots represent genes that are not differentially expressed between the two species.

The 100-bp paired-end Illumina sequence reads from *B. burgdorferi* B31 (82,056,756 reads) and *B. garinii* SZ (145,680,918 reads) libraries were combined in a *de novo* assembly to obtain the final unigenes (89,827,575 bp) and 65,535 good quality contigs. Approximately 43.7% of transcripts (n = 28,610) mapped to the Swiss-Prot database (E < 10^−4^) based on deduced amino acid similarity. A total of 1,347 and 1,454 genes were generated for *B. burgdorferi* B31 and *B. garinii* SZ transcriptomes, respectively. Sequence reads mapped against the final unigenes were used to quantify gene expression levels based on the number of reads per kilobase of coding sequence per million mapped reads. On a more conservative level using Fisher’s exact test (false discovery rate < 0.05) and fold-change ≥ 2, a total of 731 genes were differentially expressed between *B. burgdorferi* B31 and *B. garinii* SZ isolates, with 288 genes upregulated and 443 genes downregulated in *B. garinii* SZ (Additional file 1: Tables S1 and S2 and Figure 1).

**Figure 2:**
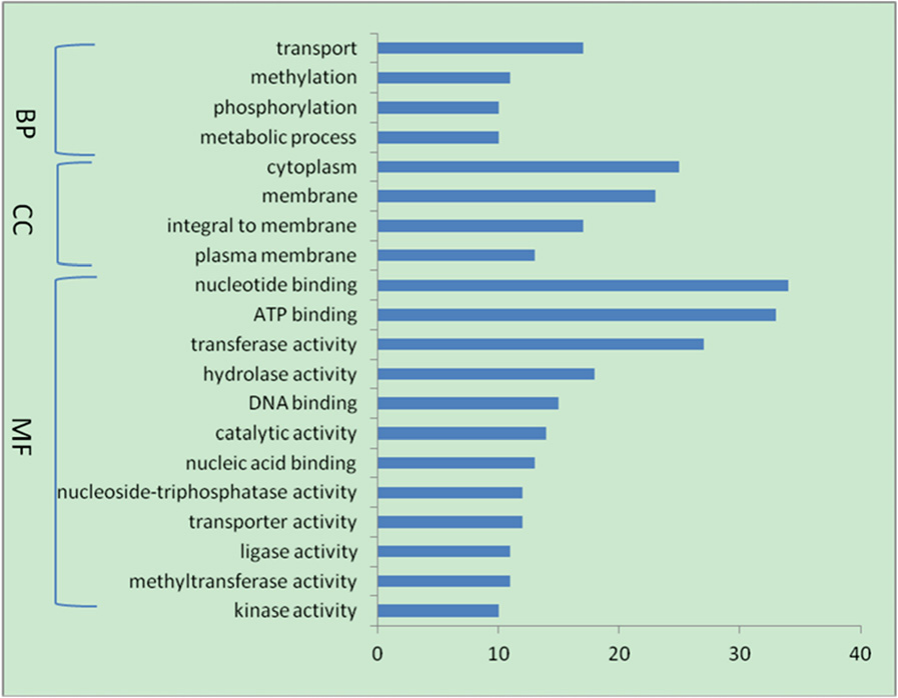
**Functional categories of genes upregulate in B.garinii SZ.**

**Figure 3:**
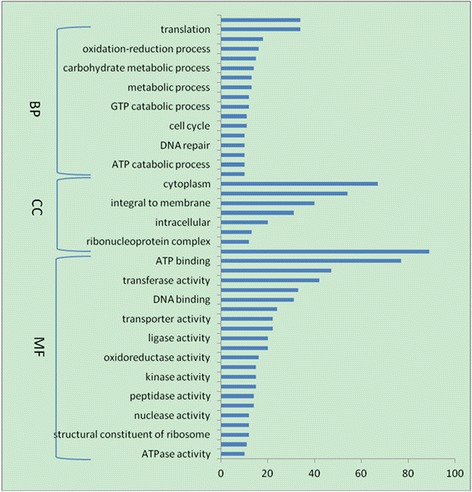
**Functional categories of genes upregulated in** ***B. burgdorferi*** **B31.**

To determine the functional significance of differentially expressed genes, BLAST2GO was used to examine the associations between GO and biological function. A total of 264 genes mapping to GO terms were identified among upregulated genes (Additional file 1: Tables S3 and S4), as well as 439 genes mapping to GO terms among downregulated genes; of these, 343 were classified as having a molecular function, 306 were implicated in biological processes, and 71 encoded cellular components. When the analysis was restricted to genes with putative biological functions, the number of genes differentially expressed between the two isolates was consistently higher in all functional categories (Figures 2 and 3). Genes that were the most highly upregulated in *B. garinii* SZ were those encoding membrane-associated proteins (20.8%) and proteins with ATP-/nucleotide-binding function (23.2%). The most highly upregulated genes in *B. burgdorferi* B31 encoded cytoplasmic proteins (13.6%) and proteins with ATP-/nucleotide-binding function (18.2%). The KEGG pathway analysis revealed that 288 of the genes that were upregulated and 443 of the genes that were downregulated in *B. garinii* SZ could be assigned to one or more of 52 and 80 KEGG pathways, respectively (Additional file 1: Tables S5 and S6).

### Lipoproteins

A large fraction of the *Borrelia* genome encodes lipoproteins such as the well-studied outer surface proteins (Osp). Several *Borrelia* proteins have been identified that interact with either host or tick ligands and thereby promote pathogen survival [17], which is facilitated by the differential expression of specific genes at various stages of the *Borrelia* infection cycle. This is best exemplified by the up-/downregulation of OspA–F, DbpA or B, multicopy lipoprotein (Mlp)-8, RNA polymerase sigma S, OspE/F-related proteins (Erp), and OspE/F-like proteins during tick feeding, transmission, and infection [18-22]. Differentially expressed genes with the highest levels of expression in *B. burgdorferi* B31 and *B. garinii* SZ are shown in Tables 1 and 2. The majority of genes encoded membrane proteins, which have important antigen-related functions. *B. burgdorferi* gene expression in the host are influenced by humoral and cellular immunity factors [23]. Complement regulator-acquiring surface proteins (CRASP) [24] and Erp bind factor H or four-and-a-half LIM domain protein, thereby inhibiting complement-mediated bactericidal activity [25]. The ability to inhibit complement varies between *Borrelia* genospecies: Erps have different affinities for factor H proteins from various animal hosts [26]. In the present analysis, CRASP and ErpA/D/P were upregulated in *B. burgdorferi* B31, while ErpY was upregulated in *B. garinii* SZ, indicating that the expression of Erp subtypes is species-specific (Additional file 1: Tables S1 and S2), which may cause different humoral and cellular immune responses in the host and contribute to the genotypic variation of *B. burgdorferi* in the pathogenicity of Lyme disease. This supports the hypothesis that global changes in gene expression underlie differences in *Borrelia* pathogenicity.

**Table 1:** **Genes with the highest transcript levels in** ***B. garinii*** **SZ**

**Locus**	**Gene**	**Description**	**Log**_**2**_ **(fold change)**
G0IT27	Erf	Erf superfamily protein	26.27
G0ANB6	Mlp	Mlp lipofamily protein	24.67
B8DXK7	OspE	Outer surface protein E	22.66
B8F1A2	OspD	Outer surface protein D	21.23
K0DGB8	BmpD	Basic membrane protein D	22.49
Q0SLT0	P13	Borrelia membrane P13 family protein	21.61
B9X9C0		Rev protein	21.00
C0R6G3	ErpY	ErpY protein	20.27
D5BEM4	Omp121	Outer membrane protein Omp121	18.77
K0DGT1	P66	Membrane-associated protein P66	11.96
E4QH49		BBD14-like protein	19.83
K0DF80		NifS protein	19.91

**Table 2:** **Genes with the highest transcript levels in** ***B. burgdorferi*** **B31**

**Locus**	**Gene**	**Description**	**Log**_**2**_ **(fold change)**
Q547V1 G8W6T3	DbpA/B	Decorin binding protein B DbpA/B	24.49
Q8KKG6	OspA	Outer surface protein A	23.19
C6C2K1	OspB	Outer surface protein B (OspB)	14.11
E4S1K9	BmpA	Basic membrane protein A	25.06
Q9S036	ErpP	Complement regulator-acquiring surface protein 3 precursor protein ErpP	26.06
O50951	P27	Surface lipoprotein P27	25.84
O51398	YidC	Membrane protein insertase YidC	23.85
E4QEQ3	FliF	Flagellar M-ring protein FliF	27.62
O51576		Uncharacterized protein	30.99
E4S2W7		Putative uncharacterized protein	28.74

### Purine transport proteins

The uptake of preformed purines by spirochete represents the first step in the purine salvage pathway, which is critical for the infection of mammalian hosts by *B. burgdorferi*. The genes *bbb22* and *bbb23*, which are present on circular plasmid 26, encode key purine transport proteins that are essential for hypoxanthine, adenine, and guanine transport [27], while inosine-5′-monophosphate dehydrogenase (encoded by *GuaB*) and guanosine monophoshpate synthase (encoded by *GuaA*) are two key enzymes in the purine salvage pathway [28]. GuaA and B were significantly upregulated in *B. burgdorferi* B31 as compared to *B. garinii* SZ (Additional file 1: Tables S1 and S2). Genes encoding bifunctional purine biosynthesis protein (PurH) and non-canonical purine nucleoside triphosphate (NTP) pyrophosphatase were also identified. These findings suggest that this transport system is a potential target for antimicrobial agents in the treatment of Lyme disease.

### Confirmation of RNA-seq data by qRT-PCR

**Figure 4:**
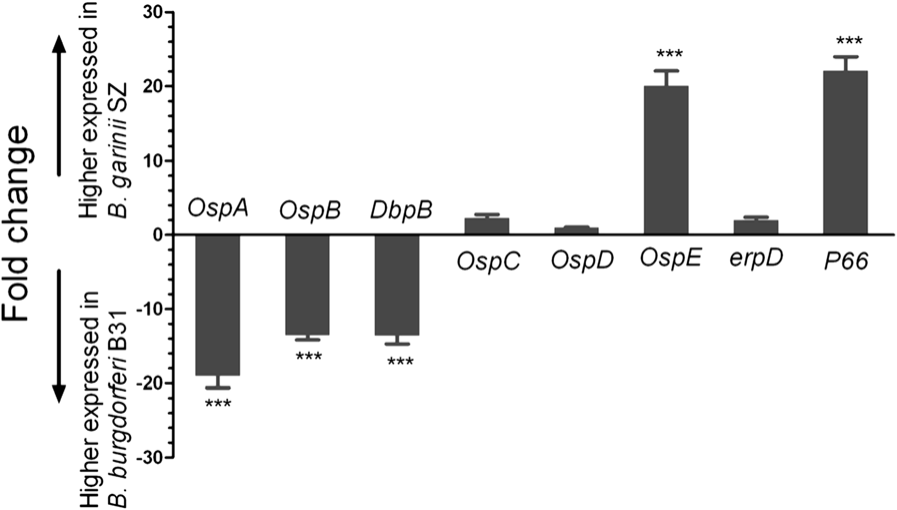
**Expression profiles of genes encoding** ***Borrelia*** **membrane proteins detected by qRT-PCR.** Each bar represents the fold change of gene expression in *B. garinii* SZ vs. *B. burgdorferi* B31. Expression levels were nomalized to that of *fla*B, and levels in *B. burgdorferi* B31 were used to calculate fold change based on a mean of three biological replicates. Bars above and below the x-axis show genes that are up- and downregulated, respectively, in *B. garinii* SZ. ΔΔCt values were analyzed with the Student’s *t* test. *P < 0.05, **P < 0.01, ***P < 0.001.

The expression levels of select genes—particularly those encoding lipoproteins and/or surface proteins—were confirmed by qRT-PCR. RNA isolated from *B. burgdorferi* B31 and *B. garinii* SZ isolates had an A_260_/A_280_ between 1.8 and 2, indicating high purity, and the PCR efficiency for each primer set was below 0.1 (Additional file 1: Table S7). The fold difference in expression levels for *B. garinii* SZ vs. *B. burgdorferi* B31 ranged from 22.07 to 1.01 (Figure 4). There were no differences between the two species in *OspC*, *OspD*, and *erpD* expression; however, *OspA*, *OspB*, and *DbpB* expression levels were significantly lower in *B. garinii* SZ than in *B. burgdorferi* B31. DbpA and B bind to the host extracellular matrix component decorin [29], and decorin-deficient mice infected with *B. burgdorferi* show reduced *Borrelia* numbers and less arthritis than infected wild-type controls [30]. The high expression of *DbpA* and *B* in *B. burgdorferi* B31 may be associated with arthritis severity and could explain the wide global distribution of this genospecies. OspA/B function was not required for infection of mice or accompanying tissue pathology, but was essential for the colonization of and survival within tick midgut by *B. burgdorferi*, events that are critical for sustaining its natural enzootic life cycle [31].

Given the increasing incidence of and medical concerns related to Lyme disease, vaccination, drug treatment, and pathogenic mechanisms have received considerable attention. Some insight is gained from the study of other faculative pathogens such as those responsible for cholera and malaria using high-throughput cDNA sequencing techniques on organisms grown in laboratory medium or isolated from infected hosts [11,32]. Thus, RNA-seq-based transcriptome analyses of pathogens during infection offer robust, sensitive, and accessible datasets for evaluating regulatory mechanisms driving pathogenesis [33].

## Conclusions

The availability of fully sequenced genomes offers new opportunities to identify genotype–phenotype relationships and undertake global genomic, proteomic, and transcriptomic analyses to investigate the biological significance of paralogous gene families and other unique features of genomes. The present study is the first to characterize the transcriptome of *B. burgdorferi*, the causative agent of Lyme disease. Some novel genes, including a bifunctional PurH and non-canonical purine NTP pyrophosphatase, were also identified that could potentially be targeted by antimicrobial agents for disease treatment. Moreover, the differential expression of specific factors observed between *Borrelia* genospecies could explain the variation in disease pathogenicity. These findings provide a framework for future studies examining the molecular mechanisms underlying the pathogenicity of Lyme disease.

## Additional file

Additional file 1:
**Tables S1.** Using Fisher’s exact test (false discovery rate < 0.05) and fold-change ≥ 2, with 288 upregulated genes in *B. garinii* SZ. **Tables S2:** Using Fisher’s exact test (false discovery rate < 0.05) and fold-change ≥ 2, with 443 downregulated genes in *B. garinii* SZ (upregulated in *B. burgdorferi* B31). **Tables S3.** A total of 264 genes mapping to GO terms were identified among upregulated genes in *B. garinii* SZ. **Tables S4.** A total of 439 genes mapping to GO terms among downregulated genes in *B. garinii* SZ (upregulated in *B. burgdorferi* B31). **Tables S5.** The KEGG pathway analysis of the genes that were upregulated in *B. garinii* SZ. **Tables S6.** The KEGG pathway analysis of the genes that were downregulated in *B. garinii* SZ (upregulated in *B. burgdorferi* B31). **Tables S7.** The primer and the PCR efficiency for each selected genes.
