# Supplementary material for: RNA-Seq-based analysis of changes in Borrelia burgdorferi gene expression linked to pathogenicity
Source: Parasit Vectors. 2015 Mar 13;8:155. doi: 10.1186/s13071-014-0623-2 (PMC4395869; doi:10.1186/s13071-014-0623-2)
Supplement: Additional file 1: — Tables S1. Using Fisher’s exact test (false discovery rate < 0.05) and fold-change ≥ 2, with 288 upregulated genes in B. garinii SZ. Tables S2: Using Fisher’s exact test (false discovery rate < 0.05) and fold-change ≥ 2, with 443 downregulated genes in B. garinii SZ (upregulated in B. burgdorferi B31). Tables S3. A total of 264 genes mapping to GO terms were identified among upregulated genes in B. garinii SZ. Tables S4. A total of 439 genes mapping to GO terms among downregulated genes in B. garinii SZ (upregulated in B. burgdorferi B31). Tables S5. The KEGG pathway analysis of the genes that were upregulated in B. garinii SZ. Tables S6. The KEGG pathway analysis of the genes that were downregulated in B. garinii SZ (upregulated in B. burgdorferi B31). Tables S7. The primer and the PCR efficiency for each selected genes. [file 13071_2014_623_MOESM1_ESM.zip › Table S7.doc]

Supplementary Table 7.

| Name | Primer Sequence | Tm (°C) | Efficiency^a^ | Efficiency^b^ |
| --- | --- | --- | --- | --- |
| *ospA*-F | 5’- GGT CAA ACC ACA CTT GAA GTT -3’ | 56.06 | 1.10 | 1.05 |
| *ospA*-R | 5’- GTC AGT GTC ATT AAG TTC -3’ | 50.47 | 1.10 | 1.05 |
| *ospB*-F | 5’- TGAGTATTCAAGTGTAGTTCCGTT -3’ | 56.84 | 0.85 | 0.81 |
| *ospB*-R | 5’- GGTTCAAAGCCTGACAAGAG -3’ | 57.80 | 0.85 | 0.81 |
| *ospC*-F | 5’- GCC GTG AAA GAA GTT GAG ACC TTA -3’ | 60.26 | 0.96 | 1.03 |
| *ospC*-R | 5’- TAA GAT TGT CCA GAC CAA GCA CTG -3’ | 60.26 | 0.96 | 1.03 |
| *ospD*-F | 5’- TAT CTT GTG TTC ATG ATA AAC -3’ | 50.20 | 0.83 | 0.90 |
| *ospD*-R | 5’- ATC CAA TGT CTC TTT GAC TG -3’ | 53.70 | 0.83 | 0.90 |
| *ospE*-F | 5’- GCC ATC TTC TTC TTT TCT TA -3’ | 51.65 | 0.86 | 0.85 |
| *ospE*-R | 5’- AGG TAT TGC ATT ATC TTT GA -3’ | 49.60 | 0.86 | 0.85 |
| *P66* -F | 5’- AAC ATC TAT CGG TCT TTA CG -3’ | 53.70 | 1.04 | 0.96 |
| *P66*-R | 5’- AAA TGT CAG ATT AGG GGT TG -3’ | 53.70 | 1.04 | 0.96 |
| *erpD*-F | 5’- GCG ATG ATC CTA ATG GCA-3’ | 55.02 | 0.82 | 0.75 |
| *erpD*-R | 5’-CCA AGT TGT ACG TAT AGA GCT-3’ | 56.06 | 0.82 | 0.75 |
| *DbpB*-F | 5’-CTG GAA ACA GTG GTC AAT TC-3’ | 55.75 | 0.96 | 0.86 |
| *DbpB*-R | 5’- TCT CTC CAC CAT TTT CAA T-3’ | 51.09 | 0.96 | 0.86 |
| *flaB*-F | 5’-GTG CAT TTG GTT ATA TTG AG-3’ | 55.65 | 1.13 | 1.10 |
| *flaB*-R | 5’-CAG ACA GAG GTT CTA TAC A-3’ | 55.02 | 1.13 | 1.10 |

^a^ Efficiency in *B. garinii* SZ was calculated by the equation *E* = 10^[-1/slope]^-1.

^b^ Efficiency in *B. burgdorferi* B31 was calculated by the equation *E* = 10^[-1/slope]^-1.
